# Illustrations of the Theory and Practice of Ventilation, &c.

**Published:** 1844-07-01

**Authors:** 


### Medico-Chirurgical Review,

### No. LXXXI.

[No. 41 of a Decennial Series.]

APRIL 1, TO JULY 1, 1844.

I. Illustrations of the Theory and Practice of Ventilation, &c. By *David Boswell Reid*, M.D. F.R.S.E. London. 1844.

II. Thermal Comfort: or, Popular Hints for Preservation from Colds, Coughs, and Consumption. By Sir *George Lefevre*, M.D. Second Edition. London. 1844.

We take it that the increased attention which has of late years been paid to both personal and public health is a proof that the medical profession frequently acts in a very disinterested manner. We are continually occupied in lessening the possibility of the occurrence of those very maladies, whose existence is a source of emolument to us. It is quite true, that if medical men banded themselves together for the purpose of preventing the removal of the causes of disease, just as various monopolizing fraternities do for the limitations of the necessaries and luxuries of life, their conduct would be met with the indignation of the public, not because it would be more injurious or destructive to life and happiness than the proceedings of the worthies alluded to, but because its ill-effects, although not greater in extent, would be more obvious in operation. A state of apathy or inaction, however, might have been tenable with little or no reproach, the general feeling with the world at large being that if it is not allowable that a man should use his power in injuring, by impeding the flow of benefits to, others, so is it not to be expected of him that he should go out of his way to benefit them, by injuring his own interests. Medical men take higher ground than this: and we may say, with truth, that the benefit they confer on society, by relieving the various diseases it has become afflicted with, is well nigh equalled by that derived from their counsels for the prevention of their recurrence or propagation. A practitioner is consulted for some ailment, which, perhaps, he is successful in relieving: but what reflecting patient does not perceive the great incidental advantages he has derived from the intercourse he has held with his adviser, whether these relate to a prevention of a return of the same malady, or to general observations upon the preservation of his health or that of other members of his family. So in medical books, a leading feature is always the consideration of the prevention of the diseases they treat of, and that frequently, not by the agency of the class to whom the works are addressed, but by modes too simple to require their aid, and yet not sufficiently obvious until pointed out. In fact, the general tenour of professional feeling has always taken a high moral tone; and has discouraged all attempts at concealment and mystery, even in the treatment of disease, and has encouraged, and often powerfully urged, the adoption of efficient preventive means. Almost every improvement in the sanitary condition of the public has been suggested by the observation and philanthropy of medical men, whose education and opportunities, indeed, furnish them with illustrations of the importance and necessity of change in various practices long before these are perceived by even the observant portion of the public.

There has always been a considerable class of publications dedicated by medical men expressly to furnishing instruction upon the preservation of health; and at no time have these been so prolific, or so well-written, as in our own. Every circumstance of public and personal hygiene has been subjected to long and repeated examinations; and the instructions deduced from these have become now, to a considerable extent, practically useful. The management of health, in every stage of life, has been placed upon a more sure basis, the occurrence of disease rendered less frequent, and, so to speak, less excusable, and its removal more easy and certain. Much remains to be done: but the great point is to rouse public attention to the necessities of the case, and this, we think, has been to a considerable extent accomplished.

Although Dr. Reid's "Illustrations of Ventilation" does not fall exactly within the category of works we have alluded to, and embraces in its exposition of the exemplifications of the subject, a much wider sphere; yet, it is especially important in the point of view we have mentioned, viz. in calling attention to the evils it would remove, and showing to how much greater extent they prevail than is generally supposed. How far the means here detailed have been successful in the gigantic undertaking of ventilating and warming the Houses of Parliament, or how far the objections hold good to the plans suggested, and, we believe, as yet not very satisfactorily put into operation, for ships, &c. we are not in a condition to offer an opinion; but of the soundness of the principles laid down, of the value of the directions given, and the importance of the exposure of the general defective condition of ventilation among us, we entertain no doubt whatever, and think the public is much indebted to Dr. Reid for the able, and yet easily understood, manner in which he has developed them.

We need not urge upon the medical reader the importance of an abundant supply of pure air. Time was, when this might have been necessary; but the woeful experience of over-crowded hospitals, &c. has produced a complete unanimity of opinion upon this subject among the profession. Some of Dr. Reid's calculations of the aggregate quantity required by large masses of people are interesting. Before quoting these, it may be observed, he considers a much larger quantity of air is required for healthy respiration than many other persons do. Thus, while different observers state two, or four cubic feet of air per minute, to be requisite, Dr. Reid places the quantity, under ordinary circumstances, as high as ten feet.

"If we look to the fact that less than half a cubic foot of air passes through the lungs of an adult in a minute, this estimate may at first appear excessive: but if we remember that at each expiration, a quantity of air is emitted which mingles with an additional portion of air largely exceeding its own bulk, and that there are twenty such respirations in a minute, while provision is likewise required for the air that affects the surface of the body, and for the endless variety of minor effects produced by furniture, lighting, heating, refreshments, &c. where no peculiar adaptations for these purposes have been introduced beyond those usually observed, it will be seen that the estimate is by no means immoderate. The real question is not what the constitution can bear, but that amount which is conveniently accessible in ordinary habitations, and which is essential for the wants of the system."

The following calculations are founded upon the mean of various observations by different experimenters.

"The inhabitants of London, amounting to two millions, respire every minute 370,370 cubic feet, or 12½ ton of air, and consequently require for respiration alone 6,653,000 tons per annum. Allowing, however, 10 cubic feet per minute to each individual for the supply of his various wants, the consumption amounts to 359,000,000 tons annually, or nearly 1,000,000 tons daily. They evolve from the lungs about 220,000 tons of carbon, and 215,000 tons of water. * * * In a room 12 feet square, and 12 feet high, containing therefore 1728 cubic feet of air, there are ten persons who respire the whole air in the room in 15½ hours, and require a complete change every 17 minutes in order to supply them with the 10 cubic feet per minute. * * * One man during a life of 50 years makes 525,600,000 respirations, inspires 166.3 tons of air, consumes 18.57 tons of oxygen, discharges 19.8 tons of carbonic acid, containing 5.475 tons of carbon, or about 80 times the weight of his own body (150 lbs). * * * The inhabitants of the earth, taken at 1,000,000,000, respire annually 3,327,000,000 tons of air, and evolve 109½ million tons of carbon. The total weight of the atmosphere is about, 5,261,000,000,000,000 tons, so that it would require 1,580,000 years to elapse before the whole atmosphere could be respired by the human inhabitants of the earth. * * * The whole inhabitants of the earth discharge annually from the lungs 107,000,000 tons of water, a quantity which, if collected together, would form a sphere nearly 2000 feet in diameter." 16.

The quantity of air required varies much under different circumstances. There is a certain amount which, if not furnished, death ensues, but above this there is every gradation until a complete supply is attained, which, in cold weather, is attended with a difficulty and expense that place fresh air amid the luxuries of life. The perception of the impurity of air and the necessity of change becomes blunted by habit, and thus the air of a coal mine, which will not support the combustion of a candle, but only that of a lamp, and which may cause fainting or death to one unaccustomed to it, is breathed by the miners with impunity. So ill-ventilated apartments which produce an oppression of the respiration to one first entering them from a purer air, do not produce this effect upon those who are habituated to them.

Where numbers are crowded together, much air is required, not only for the maintaining the respiration, but also for the sake of diminishing the augmented heat. Air entering a crowded apartment at 60° at the floor, may acquire a temperature of 70° or 80° before it reaches the head ; and Dr. Reid has often noticed a difference of 11° and 12° in winter between the temperature of the floor of the lower deck of a man-of-war, where the air was permitted to enter, and that of the atmosphere about five feet above on the same deck, where the hammocks were slung. Even in a dense crowd in the open air, on a dull or calm day, when no current of air is produced by the sun's rays, the atmosphere becomes perceptibly offensive and impure.

Upon the effects of a free or restricted supply of air upon the appetite for food, &c. Dr. Reid thus observes :—

"It appears to be universally admitted, that a low diet diminishes the necessity for much air, and that, on the other hand, where there is little air, there cannot be a great appetite for food. There are no periods accordingly, if we except a period of severe bodily exercise, where the constitution demands such a variety of supply as immediately before and after dinner; and, in the present state of society, a large share of the evil not unfrequently attendant upon a dinner party, does not always arise so much from individuals having taken more than their constitution requires, but rather from the vitiated air with which the system is usually surrounded at such periods."

In illustration of this opinion a circumstance is related of a large party of Edinburgh philosophers being enabled to unconsciously consume many times more than their ordinary complement of wine, without any immediate or ultimate ill-effects, in consequence of the dinner being given in a properly-ventilated saloon. So, too, it is said, men have struck for wages in those manufactories where efficient ventilation has been established, in consequence of being unable to obtain the additional amount of food their improved appetites demanded. If this be not an exaggeration we fear it offers little encouragement to augment the means of ventilation in the abodes, &c. of the humbler classes. People who have the greatest difficulty in obtaining the wherewith to satisfy the cravings of hunger already, have little need of the additional torture of a keener appetite. Indeed, in another part of his work, the author allows that fresh air must not be too freely introduced into the abode of misery, as there are other still more pressing and incompatible wants.

"Ventilation need not be expected where food, fuel, and clothing are deficient. Heat is still more essential to the human frame than fresh air, which consumes the body by slow combustion or oxygenation, when food is not supplied. Defective ventilation reduces the oxygenation, preserves warmth, stupifies the feelings, and allays the pangs of hunger. By reducing the power of oxygenating the blood, and of sustaining the temperature, it produces a condition which diminishes the relish and power of digesting plain and wholesome food. Unnatural stimuli, such as ardent spirits and opium, are required to excite the languid circulation, and make it feel, though only temporarily, that vigour of circulation which gives animation and vivacity to the intellect, as well as strength to the body. The actual state of the atmosphere, in the habitations of the extremely poor, is too bad and too revolting to be understood by any except those who actually visit them. There are unquestionably many exceptions, but in general, external cleansing, and an improved condition in their means and habits are essential, before any decided improvements can be extensively introduced. If draughts and currents are offensive to all, there are few who are more sensitively alive to them than those whose hunger, want of clothing, and want of fuel, reduces them to the lowest scale of misery." 40.

However unavoidable this may be with the destitute, there is another class of the community who turn foul air into current coin.

"Nowhere is the operation of a vitiated atmosphere seen more distinctly than in numbers of the refreshment-rooms in which this great metropolis abounds. Many a hard-worked clerk too often imagines he has had enough for his support, because he has taken all that his appetite permitted; whereas the saturated atmosphere in which he dines may have reduced his appetite by a half, and made him contented with an inadequate supply. The profits in numerous such rooms, would diminish largely were they well ventilated, and were the appetite equally satisfied at the same time. But the subject is as yet very imperfectly known, and escapes, in a great measure, at present, the attention of both parties. Unquestionably, however, the landlord or proprietor of ill-ventilated refreshment-rooms has no right to charge at the same rate as those that have their apartments well ventilated." 182.

Speaking from former experience, we are inclined to think that the amount of food taken by the frequenters of the metropolitan eating-houses is amply sufficient for the inhabitants of cities, leading, as they for the most part do, comparatively sedentary lives; and as to the price, it seems surprising how so substantial a meal of good wholesome food can be furnished at so low a cost. We do not deny that a complete ventilation might much increase the appetite and the quantity of food taken, and the extent to which these both occur in the purer air of the country may be adduced; but we believe that, except among the poorest classes, the inhabitants of large towns already consume larger quantities of food than the demands made upon their system by habits of activity justify.

The air may be contaminated by various sources of impurity, especially in large cities, where these so extensively prevail; but the principal one and that which most have to contend against is the spoiling it by substituting carbonic acid gas for the oxygen removed in respiration, combustion, &c. &c. This substance, existing in the atmosphere as one part in two thousand is not to be looked upon in the light of an impurity, sufficing then only for the due supply of carbon to the vegetable kingdom. It is rare, however, to meet with air of this purity in dwelling-houses, and a far larger proportion is borne even with impunity. This has its limits.

"Some individuals work habitually in mines and other places, where from one to two per cent. and perhaps still larger quantities of carbonic acid are present. But death has ensued, in some peculiar cases, it is affirmed, where there was only one per cent. Any ordinary atmosphere containing one per cent. of carbonic acid must be regarded of very inferior quality, and not fit to sustain health, though in numerous apartments a much more vitiated air may be observed. Air containing 10 per cent. of carbonic acid, and other impurities of less note, a considerable amount of moisture, and at the same time a high temperature, constituted the most impure atmosphere charged with carbonic acid to which I have been exposed; nor am I aware that I could have resisted its action for any considerable period, had its influence not been gradually developed, as the pure air in which the experiment began had successive quantities of carbonic acid communicated to it.

"When carbonic acid is present in smaller proportion than is necessary to produce an immediate and overwhelming effect, it induces a trifling headache or intolerable oppression in endless gradations, according to constitutional peculiarities, and different circumstances connected with the state of the atmosphere in which it acts." 200.

It is an error to suppose that carbonic acid gas, by reason of its greater specific gravity, is found at the lower part of an apartment or locality in which it is generated. It is only when in large quantities and in a concentrated state that it descends like water, and accumulates in cavities on the surface of the ground. It is thus found in large graves, in old wells and pits, in brewers' vats, in the Grotte del Cane, &c.; but by reason of the property of diffusion which gases of different specific gravities, brought in contact with each other, possess, no gas, however heavy, remains for any length of time at the surface of the earth, and thus carbonic acid gas has been found at the highest altitude yet reached by man. The superior heated or rarified condition of respired or burned air causes it to ascend to the highest point in the apartment which is accessible to it, and thus it is at the upper parts of rooms, &c. and not below, where the fresh air enters, that the greatest amount of impurity exists.

If the apartments of private houses are insufficiently ventilated, public buildings, in which large numbers of persons occasionally assemble, are notoriously so. The supply of pure and the removal of vitiated air has hitherto formed no part of the architect's study; and, now that public attention has become roused to the necessity of this, alterations in buildings have to be effected at a great cost and in an imperfect manner, to produce the same effect which might have been provided for in the original construction of the building at little or no additional expense. The author signalizes churches as the buildings par excellence defective in ventilation, many of these containing no provisions whatever for change of air, although much crowded, and in the evenings adding to the impurities and consumption of oxygen by powerful illumination.

"In those churches in which I have watched the progress of vitiated air, as the service proceeded, on individuals whose constitutions were not previously rendered dead to the influence of pure air by the atmosphere they were accustomed to at home, a slight and marked flush in the countenance usually appeared in a short time: this was soon succeeded by a sense of heat and oppression, and a tendency to sleep, more or less marked according to the condition of the atmosphere, and the extent to which the attention was engaged. The soporific influence was in some cases a source of annoyance to the conclusion of the service, but was succeeded in others by a re-action. The *vis medicatrix naturæ* did not remain inactive. The pulse rose, the circulation was accelerated, the brain became stimulated, and relief was at the same time afforded by the insensible perspiration (which had been arrested by the state of the air) becoming gross and sensible. Attention could now be sustained, but headach more or less severe was the usual consequence, and liability to dangerous colds and rheumatisms, where the body was suddenly exposed to the chilling influence of the external atmosphere, while still affected in the manner described."

These ill effects were always found, in a long series of examinations, most considerable where the proportion of carbonic acid gas was greatest. No means moreover are adopted to ventilate the church from week to week, or between the services, so that the congregation of the later part of the day is breathing the foul air vitiated by that which preceded them; and those of them who sit in the galleries receive only the air already contaminated in the area.

Schools, again, of both rich and poor, but of course especially of the latter, are lamentably ill supplied with air, which must powerfully contribute to the production of ill-health among the young, who so particularly, during the condition of growth, require an efficient oxygenation of the blood.

To secure due ventilation it would seem to be sufficient to provide means for the escape of air as fast as it is vitiated, the means of ingress being usually sufficiently abundant in ordinarily-constructed abodes to allow a sufficiency to enter to supply the place of that which is removed. In this way a continued movement of the air is produced, without which no ventilation can ensue. The slightest elevations of temperature are sufficient to induce such movement, by rarifying and causing the ascent of the air nearest the heated body, the colder surrounding air rushing in to supply the vacuum thus formed. The atmosphere at a mean temperature and not agitated by wind, is warmed and expanded in this way (to about five degrees) on coming in contact with the human frame, and thus a current of air is constantly passing around it. The law of this motion of the atmosphere is thus stated. "The expanded warm air being always pressed upwards by any colder and denser air, and the movement being more or less rapid, according to the difference of temperature and amount of expansion between the air that is heated and the cold air in contact with it." Ventilation, however, at most seasons of the year by no means consists in the simple admission of the external air, and expulsion of that which is vitiated. At all periods, the latter should be provided for, but the difficulty consists in the colder portions of the year as to the admission of the former—a due supply of warmth being as essential to health and comfort as a due supply of air. Numerous have been the attempts to combine the effectual warming and ventilation of apartments, but the success which has attended them has been by no means commensurate with the attention bestowed and expense incurred.

The ventilation and warming of an ordinary English apartment by means of the grate and open chimney has been condemned by all as ineffectual, partial, and expensive; and those who have suffered from the inequalities of temperature, the currents of air, and the long coal-merchant's bills incidental thereto, must acknowledge that the objectors have reason on their side. It is familiarly known that, while a person seated near a good fire is suffering from an excess of heat reflected upon his person in one direction, he is subjected to the impinging in another of currents perhaps ten or twenty or more degrees too cold: and that, while one individual a few feet from the fire place may be comfortably warm only, another as many yards may be suffering from cold. The large proportion of the heat generated which is wasted in passing up the chimney needs no demonstration. With this loss of caloric even ventilation is imperfectly attained. The heat of the fire it is true determines the upward movement of the air in contact with it, whose place is supplied by other portions, and the motion so produced causes a certain amount of ventilation. But the air which has been vitiated rises by its lesser specific gravity to the upper parts of the room, and as the aperture into the chimney is placed much below, it cannot penetrate downwards through the mass of colder, denser air which has entered by the doors, windows, &c., and much of which passes out, little changed from when it entered, and much less requiring renewal than the stratum at the upper part of the room. Still, with all the imperfections of this mode of warming and ventilating ordinary rooms, it is the best that has been yet devised for common use; and with the alterations of making a communication from the upper part of the room into the flue, so as to admit of the removal of the light vitiated air, and of rendering the aperture of the chimney below as small as possible, as indeed is done by the register stoves, so as to prevent the needless expenditure of heat, is not likely to be improved upon.

The advocates for the use of the varieties of *stoves*, as many of the German, Russian, and our own recent modifications, triumphantly point to the equability of temperature obtainable by the agency of these instruments, and the very small expenditure of fuel they produce. Sir GEORGE LEFEVRE, in the pamphlet indicated at the head of this article, attributes the great prevalence of phthisis in this country, and the comparative immunity enjoyed by Russia from this disease, to the greater care taken in the latter country to prevent inequalities of temperature by equably warmed rooms and appropriate clothing. He dwells with great force upon the comfortable condition of the interior of a Russian house even in the coldest weather, and contrasts this with the comfortless state of most English houses during Winters of infinitely less severity, both as regards the inequality of warmth and abundant currents of air in any one apartment, and in the contrast offered by the temperature of the different ones, especially the bed-rooms, which in the Russian houses are, like indeed every part of the mansion, completely warmed.

"I have been confined to my room," he says, writing to a friend in Russia, "by an obstinate cough, to which I was always subject in the Winter season while residing in England, but which I escaped during the whole time of my residence in St. Petersburgh. I had no idea I should suffer so much from the cold, yet it is not from external cold, for I took the precaution of bringing my Russian furs with me. This was quite unnecessary; for, after all the trouble I had with them, I find that I cannot wear them here. I do not understand the reason of this apparent anomaly, for I have a thermometer in my window, and find that the same degree of cold here is much more supportable than in St. Petersburgh. I should not have dared to have ventured out without a pelisse in Russia, where I find a P. jacket quite sufficient here to keep me warm. It is certain that eight degrees Reaumur in London, are more supportable than two degrees in the imperial city. It is, however, of the indoor cold that I have to complain so bitterly, for indoor warmth is a phenomenon only to be found in such houses as are provided with stoves in the ante-room, and double windows, of which comforts, alas! I have had no experience since I left Russia.

"I am afraid you will be hardly able to read this scrawl, for I am writing to you in my bed-room, which is about the same temperature as your ice-cellars. The water is literally frozen in the jug, and I cannot see out of my windows, which are like ground-glass. * * * * It is enough to try one's temper to hear, as I do, when I complain of cold, that 'it is not so cold as in Russia'—that 'surely I ought to bear the cold better here than those who have never been exposed to severe cold.' When I come down to breakfast, with blue hands and swollen fingers, I am offered the slop-basin to dip them in. I limp as I walk—'You have chilblains, I suppose; they are common Winter guests; how dreadfully you must suffer from them in Russia!' Yet I never heard of them, or certainly never suffered from them, during ten years that I sojourned there.

"What are most annoying to me are, the constant drafts which prevail in English houses. Every gust of wind makes the casements rattle, and if the rain pelts against the windows, it penetrates through the sashes, and runs down upon the window-seat.

"The drafts from the windows are dreadful. Thus you hear of cricks in the neck from sitting upon the window-seat. It is not from above, alone, that these drafts prevail, although constant puffs of smoke do unceremoniously make their entrée. The legs and feet are also exposed to a cold air-bath from drafts which come under the doors, and which make the carpet, if it be not fastened with nails, dance up and down. The drawing-rooms are somewhat more comfortable; but then, when a party breaks up for the night, it is cruel to mount two pair of stairs to go to a miserably cold bed-room. The gusts of wind in ascending the stairs are sufficient to blow out the candle. Then, if there be no warming-pan, the dread of the cold ague-fit which awaits you between clean fresh mangled sheets, is ever before your thoughts at night. When once warm in bed, I cannot imagine how it is that human nature can muster up courage enough to leave it, considering what awaits it upon doing so, as when you hear the rap at your chamber-door in the morning—'Your shaving-water, sir,' and you must get out of bed to take it from the intruding hand. How is it that, in spite of all this, I see so many rosy faces, such colour, such health? It is all attributable to 'fresh air and cold water.'

"But then poor Amelia, where is she? Since the commencement of the cold weather her cough has much increased, and I fear she will share the fate of her sister and younger brother. I shall persuade her friends to try a winter in St. Petersburgh, and the effects of Russian warm rooms. If my cough does not get better soon, I shall myself return, for I cannot stand the cold of this climate." 19.

The means the Russians adopt to secure their constantly equable temperature are the careful exclusion of the cold external atmosphere by double doors and windows and thick walls, and the generation of artificial heat within, whose dispersion is prevented in the same way. The latter is effected by means of stoves placed in the rooms and especially in the halls, which, contrary to what prevails with us, are the warmest parts of the house. The humbler peasant caulks the spaces between the logs of which his hut is composed with moss or oakum, so as utterly to prevent air penetrating any crevice however small. His door is well closed, and his window-frames double. He has his stove in the corner of the room, the smoke escaping at the roof. The heat generated would be quite insupportable to those unaccustomed to it, and may be judged of by the fact, that "the flies, congregated in some corner, hang down like a swarm of bees, happy and buzzing in the Winter season." When the Russian quits his hot-house temperature for the external air, he covers himself with the warmest furs, and the greatest number of pelisses, &c. his means allow him to possess, or the severity of the temperature demands. His bed-room is warmer in Winter than Summer, he can undress at leisure, and finds himself amply covered by a common quilt and single blanket.

Before regretting the absence, and considering the propriety of imitating these contrivances in our own country, we must observe that it is placed in different circumstances to Russia. In Russia, a Winter of dreadful severity, and a long, unvaried, continuance of a very low temperature, are the evils to be provided against, and present death and destructive disease would prevail if ampler means of doing this did not exist there than with us. But the adoption of such means are attended with disadvantages.

The Russian, of all men, is the most susceptible of cold, owing to the hot-house temperature he accustoms himself to, and is far less able to resist its influence, and is obliged to fly to protective clothing far sooner than the inhabitant of temperate climes does at even the same temperature. Upon a large scale, the same effect is produced as with us results on a small scale, from *coddling* by excessive precaution. The least exposure unprotected is followed by serious evil, and that this is not the case in Russia arises from the care with which such exposure is carefully guarded against. Again, the impurity of the air which this system engenders is obvious. The great object of the Russian is to exclude the external air as far as possible, and he prefers continuing to respire vitiated air to the freer admission of a colder and purer atmosphere, so that among the lower classes asphyxia is by no means uncommon, and, in all, the rooms have a close, and, what we should call, unhealthy smell. It is true the rich man can have his house so constructed as to admit a sufficiency of air through his heated hall and corridors, so that it arrives in his sitting and bed-rooms in considerable quantities, but this cannot be the case with the majority. In England we have to provide against not a long-continued severity of climate, but a great variability. If, by the heated rooms of the Russians, we augmented our susceptibility to the influence of cold, we should only increase our liability to disease, seeing that we know not when a change to a lower temperature may take place, and are often unable to provide for it by artificial heat or appropriate clothing. We doubt not that the vicissitude of our climate is a leading cause in the production of consumption, but we do not believe its operation would be less prevented, but, on the contrary, increased by adopting this hot-house mode of life. More is to be even gained by accustoming ourselves to the varieties which in ordinary active life are unavoidable, and even the drafts of common dwelling-houses in this way have their advantage, and would be attended with much less evil if women, who form the majority of victims of phthisis, would cover the upper parts of their persons more completely, and their feet more warmly, than is customary. It is the neglect of this and their inactive habits that render them so extremely susceptible. Still, with the delicate and affluent, who can devote their whole attention to their health, and whom circumstances do not oblige to risk the vicissitude of the external air, when threatened by consumption, the adoption of the warm-air rooms or a visit to Russia for the Winter, where certainly house-warming is in perfection, may be desirable; and in traversing our passages the Respirator is as necessary for such as when they go into the open air. In spacious houses, having halls and passages, stoves are very useful in mitigating the change of temperature in passing through them, and might easily be rendered available for warming all the air which enters the house. But the introduction of stoves into sitting-rooms we consider objectionable, as they do not secure that ventilation which is necessary for health, and when made too warm, augment the susceptibility to cold upon exposure to it. We believe that much of the healthful appearance and superior health of the English people depends upon their free exposure to pure air; and pallid countenances and a proportionate increase of mortality are found to prevail in localities where such exposure is impossible or limited.

Upon the Fire-place, Dr. Reid has the following observations.

"The peculiar advantages of a fire-place are not merely its power of warming an apartment, the circulation of air which it induces, its accessibility, and the influence of the air which it evolves: but the very grateful effect which it produces, after the body has been chilled by any special cause, whether in-door, or out of-doors, stimulating it and exciting the circulation to the greatest degree which may be considered agreeable, and permitting each individual to adjust the distance which is most suitable to his own constitution, and the previous exposure to which he may have been more immediately subject. The light, also, is not to be considered a mere nominal advantage, but a real and positive benefit, affecting the whole system by its physical action, independently of the cheerful impression which its liveliness is calculated to excite; and which, to many, is so engaging, that they feel as if they were not alone when they have the company of a glowing fire. These considerations will probably always sustain the open fire-place, in countries where fuel can be procured with sufficient economy: but its disadvantages, in other respects, compared with the stove, are marked, particularly its expense, its local action, the dust it is apt to produce, and the frequent attendance it requires." 201.

Dr. Reid observes that, where much iron is employed around an open fire, it robs it of so much of its heat and communicates it by conduction to the air passing up the chimney, that the fire rarely burns brilliantly.

To this author's observations upon the ventilation of the House of Commons, and other large edifices, we can only refer as containing a masterly demonstration of the subject, which, owing to the profuse illustration by diagrams, (these are found, indeed, in great abundance at almost every page of the book,) becomes easily intelligible to even an unprofessional reader.

The first chapters of Sir George Lefevre's little work, treating of the benefits of greater attention being turned to warming rooms, and the employment of appropriate clothing, of the regimen and climate for those threatened with consumption, and of the value of the stethoscope, contain very useful information, calculated to be of use to the general reader. But we consider he has committed a mistake in appending the last; viz. observations upon the treatment of Pleurisy. Not but that this chapter contains matters good in themselves, but very improperly placed. This is, in fact, the error of most writers who address the public upon subjects relating to medicine; for, not content with addressing admonitions frequently invaluable as to the *prevention* of disease, and which we have alluded to with pride at the beginning of this article, they must also learnedly discourse on the treatment of diseases, and the various appliances of medicine, which a professional education is required for comprehending, much less acting upon. If attempted to be acted upon by the public, every one must see that danger must result; and if the public are expected not to meddle with them, why place them in their way. But, as we have said, although we object to comprising in a book, professing to give "popular hints for preservation from coughs, colds, and consumption," observations upon pleurisy, aqua laurocerasi, and tinct. digitalis, yet the observations themselves, are very useful, and must, therefore, be rescued from their ill-assorted company.

In the opinion expressed of the limited use of the *stethoscope* in general practice we agree, believing, that, for practical benefit to be derived from it, its use must be limited to a few consulting practitioners, possessed of frequent and constant opportunities of keeping up and improving their knowledge of its powers. In the hands of some it has been made an instrument of gross quackery and pretension.

"Constant practice can alone make perfect in this particular; and how is this to be obtained by the generality of practitioners, who have not the advantage of hospitals or large bodies of patients, upon whom they can prosecute their inquiries? Moreover, it appears to me, that a delicate sense of hearing, such as many do not possess, is a *sine qua non* for the investigation. The susceptibility to variety of sounds is not the lot of all, and those who do possess it, cannot do so at all times and under all circumstances.

"Professional mistakes, with respect to the termination of lung diseases, have been at all times common, but perhaps the public may think them less warrantable, where those means of investigation have been employed to which they attribute more importance than is really due to them: for many look upon this procedure as infallible, and elevate the instrument to the dignity of a mariner's compass. The ear may be of great use in the diagnosis of disease, and should certainly be employed in all cases where it can be fully depended upon; but we were pleased with the observation of one of the most distinguished members of the British faculty, and one perhaps more consulted than any in the metropolis, who stated to us, that he employed the stethoscope rather to confirm his preconceived opinion of a case, than to form one by this means. I am not disposed to disparage—nay, I must avow that I have witnessed the most brilliant feats in the field of diagnosis, from a thorough acquaintance with mediate auscultation, and I believe that no practitioner stands higher in reputation of this kind than Dr. Seidlitz of St. Petersburgh, the advantage of whose superior abilities I enjoyed in consultation during my residence in that capital: but, I do believe the instrument is calculated to do harm to any but adepts; and that long study, continual practice, and certain physical advantages are indispensable to employing it with advantage."

*Pleurisy, &c.*—Although the author states consumptive diseases are rare in Russia, inflammatory affections of the lungs and pleura are very common. He believes them to be more tractable than they are in our latitude, on account of the equable warmth of the apartment in which the patients are kept, preventing any currents of air; but, on account of the great contrast of the temperature of the apartments to the external air, the convalescence is a very tedious one—so that a patient attacked by serious disease at the commencement of Winter, seldom finds himself well prior to the ensuing Spring. Sir George employs bleeding in moderation, but speaks very approvingly of the German remedy, *muriate of ammonia,* in pleurisy, subacute inflammation of the lungs, and chronic congestions of the mucous membrane. The following is the formula the author has frequently employed it in, with very great advantage.

℞. Ammonia Mur. ʒj.

Ex. Glycyrrhiz. ʒiij.

Ant. Tart. gr. ij.

Aq. destillat. ℥viij. coch. 1 2dis horis.

When nausea appears, the antimony is to be withdrawn, and is often not required at all. Stomach-coughs are benefited by it; where the tongue is loaded it soon cleans it. Many affections of the mucous membranes, as sore throat, enlarged tonsils, relaxed uvula, &c. are benefited by it. For catarrhal affections, especially in children, the following is useful. ℞. Syr. Althææ ℥ij. Vin. Antimon. ʒij. Teaspoonful or two every hour or two. In aggravated cases of cough, with little expectoration and much dryness of skin, the following combination is a good one:—℞. Træ Digitalis, Liq. Ant. Tart., Aq. Lauro-ceras. āā ℥j. Dose from 30 to 40 drops, four or five times a day, in a pectoral tea prepared from various simple herbs.

The Germans have the same prejudices against the use of purgatives as the French. They employ tartar-emetic very successfully as a counter-irritant. A drachm mixed with Burgundy-pitch is spread on leather, and applied to the chest or between the shoulders. It produces intense irritation. The *aqua lauro-cerasi* is much used by the Germans, and is deservedly esteemed by them, acting as a *calmant* in spasmodic affections. The dose may be increased from 10 to 60 drops. "It is very useful in spasmodic affections of the stomach, in hypochondriac uneasiness, in hysteria, combined with pain of the uterus. Some mental affections from over-excitement are much relieved by it; I have found it very useful in calming the pain arising from the passage of inspissated bile and small biliary calculi." The following caution is worthy of imitation by us.

"The druggists in St. Peterburgh are compelled to retail all medicines which are poisonous in small doses, in dark blue bottles, and all the labels are of the same colour. A piece of paper upon which is printed 'for external use,' is pasted upon the foot of the phials containing liniments, lotions, &c."

The author concludes with the following observations upon the German therapeutics.

"German therapeutics hold a middle rank of action between the French and the English, being more energetic than the former, and less so than the latter. The Germans boast of a simplicity of prescription, and have a horror of contrarieties, carrying this to a ludicrous nicety and an unmeaning orthodoxy. Thus, a solution of sulphate of magnesia in an infusion of roses, or the combination of a laxative with an astringent, meets with the severest criticism from those who profess as much abhorrence of a contresens in prescription as Nature does of a vacuum.

"Unless in form of decoctions, which contain certainly the essence of a large proportion of the vegetable kingdom, and are favorite remedies, a German prescription rarely boasts of more than two or three ingredients. Nothing is considered inert unless it be distilled water, and if active remedies are administered in a variety of other menstrua, the latter are not chosen indifferently, but with a specific view, and to perform a part in the operation of the whole. One carminative water cannot be substituted with impunity for another, it being granted that each has a specific action upon the system. In respect to the influence of minute doses, the Germans countenance the practice of homœopathists. Their ideas (well or ill-founded as they may be) of infinitesimal doses, are illustrated in the preparation of a decoction much esteemed for the cure of eruptions which disfigure female beauty, and which is administered freely in the Spring. It is composed of sarsaparilla, dulcamara, tops of the pine, beetroot, buds of the beech-tree, &c.; but the most active ingredient is a small piece of glass of antimony tied up in a muslin bag, and boiled for a limited time only in the decoction, which is supposed to be impregnated by it, although it may have lost no weight in the operation."

The above extracts, we repeat, are interesting to medical readers, but are ill-fitted for general perusal.

